# A Stakeholder-Based Study Improving Resident and Family Engagement in the Safety of Assisted Living

**DOI:** 10.1093/geroni/igab046.1330

**Published:** 2021-12-17

**Authors:** Anna Beeber, Ruth Anderson, Lindsay Schwartz

## Abstract

Assisted living (AL), is a long-term care service that provides housing and care for over 800,000 older adults in 30,000 residences. AL culture and operations have been transforming to enhance resident personhood and increase autonomy, however, these practices are balanced with the need to minimize safety issues (e.g., medication errors, infections, falls, and in cases of dementia, elopement and injuries). In this stakeholder-based study, we are translating existing strategies for improving patient safety to AL residences and developing an evidence-based tool for implementing these engagement strategies in AL. This symposium presents the methods and findings from a federally-funded mixed methods study including qualitative interviews with 105 AL residents, staff and family caregivers, and a series of focus groups with an AL stakeholder group to develop a toolkit to improve resident and family engagement in AL safety. The first paper outlines our methodological approach, including our efforts to work with stakeholders throughout the research process. The second paper reports findings from a scoping review of existing tools to support resident and family engagement in the safety of AL. The third paper presents the findings from our interviews with AL residents, families and staff exploring their safety priorities, and how they differ across stakeholder groups. The fourth paper presents the findings from our qualitative interviews exploring the challenges and promising practice to resident and family engagement in AL safety during the COVID-19 pandemic. All four presentations in this symposium illustrate important issues for future practice, policy, and research.

